# The Changing Role of Phonology in Reading Development

**DOI:** 10.3390/vision3020023

**Published:** 2019-05-30

**Authors:** Sara V. Milledge, Hazel I. Blythe

**Affiliations:** Department of Psychology, University of Southampton, Southampton SO17 1BJ, UK

**Keywords:** theories of learning to read, orthography, phonology, adults, children, eye-tracking

## Abstract

Processing of both a word’s orthography (its printed form) and phonology (its associated speech sounds) are critical for lexical identification during reading, both in beginning and skilled readers. Theories of learning to read typically posit a developmental change, from early readers’ reliance on phonology to more skilled readers’ development of direct orthographic-semantic links. Specifically, in becoming a skilled reader, the extent to which an individual processes phonology during lexical identification is thought to decrease. Recent data from eye movement research suggests, however, that the developmental change in phonological processing is somewhat more nuanced than this. Such studies show that phonology influences lexical identification in beginning and skilled readers in both typically and atypically developing populations. These data indicate, therefore, that the developmental change might better be characterised as a transition from overt decoding to abstract, covert recoding. We do not stop processing phonology as we become more skilled at reading; rather, the nature of that processing changes.

## 1. The Changing Role of Phonology in Reading Development

Learning to read is a vital process within modern societies given how much information is conveyed by the written word, ultimately affecting academic success, employability, and social and economic welfare. For example, it is estimated that the cost of illiteracy to the global economy is over $1 trillion each year, costing a developed nation 2% of its gross domestic product (GDP), an emerging economy 1.2% of its GDP, and a developing country 0.5% of its GDP [1]. Yet the acquisition of this skill, so pivotal to successful functioning within society, is a long, complicated and effortful process that can last for many years.

Reading is a process that requires the learning of associations between the visual forms of printed words (orthography) and their associated speech sounds (phonology) and meanings (semantics). The aim of reading is to construct meaning from text, i.e., for the reader to comprehend the written language. It is well-recognised, though, that making these links from orthography to semantics also involves phonological processing [2]. Oral language acquisition precedes written language acquisition, and so, a child’s earliest cognitive representations of words include phonology and semantics; only later, as they learn to read, do those phonological and semantic representations map onto orthographic forms [3].

Within theoretical accounts of reading development, a broad consensus seems to be that as a child’s reading skill increases, their lexical identification becomes increasingly based on direct orthographic–semantic links, and the contribution of phonology to lexical identification decreases, e.g., [4,5,6,7,8,9,10]. Consequently, skilled reading is often characterised as an individual’s ability to access semantics directly from a word’s printed form. This view has been supported by data from pen-and-paper tasks, such as hand-coding of a child’s reading, spelling or pronunciation errors [11,12,13]. In recent years, though, eye movement research has indicated that children continue to process phonology during lexical identification as their reading skills increase [14]. These data indicate that developmental change in phonological processing is better characterised as a progression from early, overt decoding (the conscious, effortful sounding out of printed letters to identify a word) to more sophisticated, covert phonological recoding (the rapid, covert, pre-lexical processing of a printed word’s phonology).

We begin by briefly reviewing the literature on theoretical models of children’s reading development, which clearly documents a developmental change in phonological processing during lexical identification. We then review the literature on skilled adult readers’ lexical identification which has examined, in considerable detail, the role of phonological processing. Subsequently, research within developmental populations, both typical and atypical, is discussed. Phonological processing in languages other than English is also briefly considered (given how theories of learning to read relate primarily to reading development within English, this paper’s focus will predominantly be on research conducted in English). Finally, some models of word recognition are briefly outlined and then evaluated within the context of this paper. Taken together, we consider how these recent contributions to the experimental literature might contribute to both theoretical models of learning to read and models of word recognition.

## 2. Theories of Learning to Read

One prominent theory of how visual word recognition skills develop is Share’s [15] self-teaching hypothesis. This hypothesis posits that phonology plays a central role in how readers acquire orthographic representations of words. Phonological decoding (to achieve a correct pronunciation) is assumed to be critical for the acquisition of orthographic representations, as it draws the child’s attention to the order and identity of a word’s constituent letters. As such, decoding provides children with the opportunity to set up direct connections between the spelling of a letter string and the phonology of the spoken word, which results in the growth and development of their lexicons. In this way, phonology serves as a powerful self-teaching device: the explicit learning of a few sets of grapheme to phoneme correspondences (GPCs) allows children to decode an increasing number of words, which, in turn, supports the growth of their lexicons.

A number of theories have been proposed in order to try to characterise the process that children go through as they progress from beginning to skilled reader, with many proposing that children progress through a series of phases as they become more experienced in dealing with written text, ultimately leading to fluent, skilled reading, e.g., [5,6,7,8,9,10,16,17]. It is assumed that whilst most children pass through these phases, they are not biologically determined [18]. These phases are described as representing the reader’s dominant (but not sole) process for identifying words during reading at that point in the child’s development. There are, of course, differences between the theories of reading development. For example, some theories suggest that there are three phases, e.g., [10], while others suggest four phases, e.g., [5,6,7,8,9]. Here, we focus upon the common aspects that are relevant to our interest in phonological processing. Broadly speaking, the earliest phase(s) of reading development is characterised by a child’s attempts to learn associations between orthographic features of written text (although not complete word forms) and words that already exist in their oral vocabulary (e.g., recognising the word *camel* because it has two humps in the middle) [19]. Subsequently, children learn the alphabet and, consequently, learn grapheme to phoneme correspondences (e.g., learning that the word *cat* is pronounced */k/ /æ/ /t/*), providing the capability to read words the child has not encountered before. Then, finally, a child progresses to the point where they are able to identify the majority of printed words that they encounter through whole word recognition, with the assumption that this process relies on direct orthographic–semantic links. At this point, a child does not engage in any observable, overt phonological decoding in order to identify words during reading (for a recent review, see [4]).

A major similarity between these theories of reading development is that they propose a developmental shift from beginning readers, who rely more on phonology to identify words, to more skilled readers, who form direct links between orthography and semantics, e.g., [5,6,7,8,9,10]. Inherent in this proposed trajectory is the decreased reliance on phonology, to the point where it no longer contributes to lexical identification for most words that a reader encounters. Such theories, though, were primarily formulated on the basis of findings from offline tasks, e.g., [12,20,21]. Whilst it is true that offline tasks, and isolated word recognition tasks (as discussed in the following section), have provided researchers with insight (albeit indirect) into the role that phonological processing plays in both skilled adult and beginning child readers and in the shift from effortful phonological decoding to fluent sight word reading, e.g., [8], it is eye movement research (discussed in Section 4) with skilled adult readers, and more recently with developmental populations, that has provided direct insight into how this proposed theoretical developmental shift may be more nuanced than these current theories account for.

## 3. The Role of Phonology: Isolated Word Recognition Tasks

This section outlines four key areas of evidence: (1) delineation of how isolated word recognition tasks have demonstrated the use of overt phonological decoding by beginner readers in order to achieve lexical access; (2) how this subsequently decreases based on reading skill; (3) how adults display covert phonological recoding; and (4) the display of this form of phonological processing by children.

A substantial body of evidence has documented how readers engage in overt phonological decoding in order to identify printed words, using a variety of experimental paradigms. For example, lexical decision tasks (LDTs), where participants are required to decide, as quickly as possible, whether a printed letter string is a real word or not; semantic categorisation tasks, which require the participant to decide whether or not each presented word is an exemplar of a particular semantic category; and naming tasks, which require participants to pronounce a written letter string, often at speed, have all been used.

First, such methods have documented overt phonological decoding in beginning readers. For example, Johnston and Thompson [22] found that 8-year-old English children were less accurate at rejecting pseudohomophones (e.g., *wotch*-*watch*) than ordinary nonwords (e.g., *cotch*) in a LDT (Experiment 1). It was noted that many of the children tended to sound the stimuli out loud prior to making the lexical decision. Sounding out is a clear indication of phonological decoding being undertaken by the children, and the children displayed reduced accuracy in rejecting the nonword pseudohomophones, indicating that lexical entries were being activated for their respective “real word” homophones. Phonological decoding was enabling the children to activate an existing lexical entry due to shared phonology, regardless of the status of the pseudohomophone as a nonword (with no possible lexical entry). This tendency for children to rely on phonological decoding seems to become particularly apparent when they encounter unfamiliar words. For example, Adams and Huggins [11] selected 50 exception words, such as *ocean*, *sword* and *yacht*, which were ordered by frequency (how often a word is typically encountered in text), so that easier words preceded harder words. The researchers found that children in Grades 2–5 typically read words accurately and without any overt decoding until they reached a point in the list where the words became unfamiliar (i.e., low frequency words). At this point, readers began sounding out and blending the words, which caused them to hesitate and often misread the words. Schmalz, Marinus, and Castles [23] found that children showed regularity effects (whereby a benefit is found for regular words, that is, words with pronunciations that conform to GPC rules, e.g., *spade*, over irregular words, with pronunciations that do not conform to GPC rules, e.g., *yacht*) for low frequency words (e.g., *desk* vs. *calm*) but not high frequency words (e.g., *mess* vs. *ghost*) in a LDT. The researchers argued that children were using phonological decoding for words that they encountered less frequently because the output for irregular words from phonological decoding conflicts with the correct entry in the mental lexicon. For high frequency words, however, the lack of regularity effects suggests that children as young as 8 years-old were relying predominantly on a direct route from orthography to semantics for high frequency words.

Second, the literature shows children’s decreasing reliance on overt phonological decoding as their reading skill increases. It is posited that readers increasingly identify words by sight, with direct links from orthography to semantics, e.g., [8]. For example, Samuels, LaBerge, and Bremer [24] used a semantic categorisation task with children from Grades 2, 4 and 6 as well as college students. The words used in this task varied in length from three to six letters. Whilst second graders’ response latencies increased as words grew longer, older students’ latencies did not change as a function of word length. This suggests that the older participants were processing the words as wholes, whilst the second graders were processing component letters in order to read the words (although it is worth noting that this could be an orthographic effect rather than an effect of phonology). Nevertheless, other research has also demonstrated how phonological decoding decreases as reading skill increases. For example, Ehri and Wilce [20] measured the latencies of skilled and less skilled readers (from Grades 1–4) in a series of naming tasks using common words (e.g., *book*), number words (e.g., *four*), CVC nonwords (e.g., *jad*), and single digits (e.g., *6*). Skilled readers across the grades named words faster than nonwords and named words as quickly as digits, indicating that they were processing the words as wholes. In contrast, though, the less skilled readers only displayed this pattern of effects in Grade 4; only the oldest less skilled readers were equally as fast at naming words as digits. Overall, these data show that as children become increasingly skilled readers, decoding decreases. Researchers have often inferred from this an increasingly dominant process of direct access from orthography to semantics.

Third, a large body of evidence has documented phonological recoding in skilled adult readers. For example, Lesch and Pollatsek [25] had participants name target words (e.g., *nut*) after the presentation of a prime, either a semantic associate word (e.g., *beech*), a homophone of that associate (e.g., *beach*) or an orthographic control (e.g., *bench*). The researchers found that, at short prime durations, the target words were named faster following both the semantic associates and the homophone primes, in comparison to the orthographic controls. The researchers concluded, therefore, that phonological recoding contributed to readers’ lexical access. Van Orden [26], in a semantic categorisation task, found that frequent errors were made to homophones of particular categories; for example, for the category ‘flower’ the word *rows* is homophonic to the category instance of *rose*, and participants frequently made false positive errors to *rows* relative to orthographic controls (e.g., *robs*). As such, phonology appears to play an important role in allowing adults to achieve lexical access through phonological recoding [25,26,27,28,29].

Fourth, children have also been shown to display phonological recoding, with this form of processing seeming to be pivotal in the development of visual word recognition skills. For example, Kyte and Johnson [30] had Grade 4 and 5 children make lexical decisions for monosyllabic words (e.g., *bean*/*meat*) and pseudowords (e.g., *meap*/*meep*) under two matched experimental conditions: one where items were named prior to lexical decision to promote phonological recoding (read aloud condition), and a condition presumed to limit phonological recoding (concurrent articulation condition; participants repeated a syllable (e.g., “LA”) whilst completing the LDTs). Later, approximately 24 h after the LDTs, orthographic learning of the pseudowords was evaluated using orthographic choice, spelling and naming tasks. Target words learned with phonological recoding produced greater orthographic learning than those learned with concurrent articulation. This study provides some evidence for the importance of phonological processing in the development of visual word recognition skills and an orthographic lexicon, consistent with the self-teaching hypothesis [15]. However, it is important to note that this task requires overt phonological processing in order to name each stimulus aloud; such processing is not required in silent sentence reading. Error detection tasks have also been used to examine phonological recoding in children, where participants are required to decide whether an error is present in the context of a whole sentence. For example, Coltheart, Laxon, Rickard, and Elton [31] asked adults (Experiment 1) and children (Experiment 2) to judge whether printed sentences were correct or not. One of the unacceptable sentence conditions presented pseudohomophones (e.g., *Her bloo dress was new.*). The researchers argued that, in this condition, any observed effects of phonology must be pre-lexical because there are no lexical entries for nonwords (i.e., it is not possible for phonology to have a top-down influence, post-lexical access, as could be the case for known words). Pseudohomophone sentences resulted in significantly higher false positive rates for both adult and child readers, relative to control conditions. Thus, the authors argued that both the adults and the children were pre-lexically processing phonology (recoding). One possible caveat is that response times were not recorded, only accuracy. It is possible that readers were engaging in some form of subvocal phonological decoding in order to process the pseudohomophones.

Taken together, these studies provide strong evidence for phonological recoding in skilled adult readers, e.g., [25,26,27,28,29]. There is also clear evidence that beginning readers rely on phonological decoding and that this reduces over time as reading skill increases [20,24]. Finally, there is some evidence that once children are past the point in their reading development where they are engaging in effortful phonological decoding, they have made a transition to phonological recoding, e.g., [30,31]. Whilst these studies do suggest such a transition, they do not afford as direct insight into a reader’s cognitive processing of text as eye movement research does, especially given the offline nature of some of the data, e.g., [31]. Consequently, seeking converging evidence from different approaches could prove useful.

## 4. The Role of Phonology: Eye Movement Research

Eye movement research provides a highly sensitive index of cognitive processing during reading, affording researchers an insight into the online, moment-to-moment operations involved in the reading process [32,33,34]. As such, researchers can gain insight into the cognitive processing of text using more naturalistic sentence reading, as opposed to isolated word recognition tasks or offline tasks. A body of literature has used eye movement recordings to examine the contribution of phonological processing to lexical identification during silent sentence reading.

**Adults.** Research has strongly indicated that adults continue to make use of phonology during reading. From the literature on skilled adult reading, two roles have been proposed for phonology during skilled reading: (1) phonology may play a pre-lexical role and aid the process of lexical access and word identification; or (2) phonological codes may be activated as a function of lexical access or after lexical access [2,3].

Rayner, Pollatsek, and Binder [35] provided evidence that phonological information is activated during silent reading. Participants read short passages that contained a correct target word, a homophone, or an orthographic control (e.g., *Murderers who kill many people according to a pattern are referred to as serial/cereal/verbal killers.*). Both the orthographic controls and the homophones were incongruent with the semantics of the sentence context, and, as such, longer reading times would be expected in both these conditions relative to the correct target word. Importantly, the orthographic controls and homophones were matched in terms of their orthographic overlap with the target word. Shorter reading times on the homophone relative to the orthographic control would, therefore, be attributable to the homophone’s shared phonology with the correct target word. Strikingly, reading times on the homophone were not significantly different from reading times on the correct target word when it was orthographically similar to the target word (e.g., *heal*-*heel* vs. *right*-*write*). This suggests that readers’ early activation of congruent phonological codes resulted in the reader not even noticing that the word they were fixating was an error word (that is, a word that was incorrect in the context of the sentence). Critically, across both orthographically similar and dissimilar conditions, participants displayed shorter reading times on homophones than on orthographic controls, and this effect was observed in early measures of processing (i.e., in first fixation duration—the duration of the first fixation on a word regardless of how many fixations it receives). It is worth noting that in the researchers’ first experiment, a pseudohomophone condition (e.g., *brane*-*brain*) was also used, and the pattern of results was similar to that of the homophones. This provides further evidence for a pre-lexical role for phonology: pseudowords do not have lexical entries, so any characteristics of such words that facilitate lexical identification (i.e., shared phonology with real words) would have to be activated before lexical access is achieved [27]. Thus, phonological recoding was used by skilled adult readers in their initial fixation on a word, seemingly pre-lexically, facilitating lexical identification.

**Figure 1:**
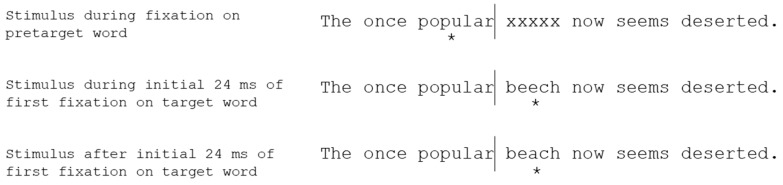
An example of the fast priming technique. The asterisk underneath each sentence indicates the reader’s fixation location. An invisible boundary is placed in a sentence in the space before a target word (the lines in the example above represent the location of the boundary, but this is not visible to participants). Before fixation, a string of xs is present where the target word should be. When the readers’ eyes cross the invisible boundary and first fixate the target word location, a prime is presented for a very brief amount of time (e.g., 24 ms), before being replaced by the target word. This example shows a homophone prime (e.g., *beech*) for a target word (e.g., *beach*).

With respect to the pre- versus lexical/post-lexical phonology question, though, the strongest evidence comes from fast priming (Figure 1; [36]) and parafoveal pre-processing studies.

Rayner, Sereno, Lesch, and Pollatsek [37] used the fast priming technique to compare identity (e.g., *beach*), homophone (e.g., *beech*), orthographic control (e.g., *bench*), or dissimilar primes (e.g., *noise*). The critical comparison here was that of reading times on the target word when it was primed by a homophone relative to an orthographic control (i.e., looking for evidence of a phonological priming effect). Participants had shorter gaze durations on a target word when it was preceded by a homophone prime than when it was preceded by an orthographic control. Thus, it would appear that phonology can be coded quickly enough to facilitate lexical access and identification of the target word. Further evidence for this argument is provided by parafoveal pre-processing studies.

**Figure 2:**
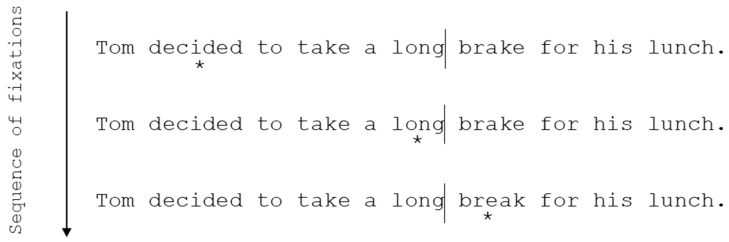
An example of the boundary paradigm. The asterisk underneath each sentence indicates the reader’s fixation location. An invisible boundary is placed in a sentence in the space immediately before a target word (the lines in the example above represent the location of the boundary, but this is not visible to participants). A preview letter string is available in the target word’s location prior to the reader making a saccade that crosses this invisible boundary. After the reader’s eyes cross the boundary, they move to directly fixate the target word. Then, a display change occurs wherein the preview letter string changes to the correct target word. By manipulating certain characteristics of the overlap (e.g., phonological similarity) between the preview string and the target word, parafoveal pre-processing can be studied. For example, phonologically consistent (e.g., *brake*) and inconsistent (e.g., *bread*) previews can be presented for a target (e.g., *break*) to examine the extent to which a reader is undergoing phonological pre-processing prior to direct fixation. If a reader does extract phonological information during parafoveal pre-processing, then reading times on the target word should be shorter following a consistent preview than an inconsistent preview. This decrease to reading times is referred to as preview benefit. If preview benefit is found, i.e., shorter reading times, on a word that was parafoveally available compared to when the parafoveal preview word was masked, this is strongly indicative of parafoveal pre-processing having occurred, as lexical identification has been facilitated. As such, parafoveal pre-processing and this paradigm enable researchers to investigate pre-lexical effects, as manipulations are conducted outside of direct fixation (i.e., lexical processing): if the manipulated characteristic of a given word in the parafovea confers preview benefit to the reader, the word must have been pre-lexically processed to some extent prior to it receiving a direct fixation.

Parafoveal pre-processing refers to readers’ extraction of information from the next word in a sentence (referred to as *n* + 1) before it is directly fixated (whilst processing is on-going for the currently fixated word- referred to as *n*). It is typically investigated using the boundary paradigm (Figure 2; [38]).

Indeed, evidence from the use of the boundary paradigm has found that phonological recoding begins prior to direct fixation in skilled adult readers. For example, Pollatsek, Lesch, Morris, and Rayner [39] found that readers can pre-process phonological cues from an upcoming word. Previews were either homophones or orthographic controls for a target word that was presented after the reader’s eyes had crossed the boundary. They found that reading times on the correct target word were shorter when the preview was a homophone than when it was an orthographic control. Such effects, indicating pre-lexical parafoveal processing of phonology, have now been shown in a number of studies looking at parafoveal pre-processing in skilled adult readers, e.g., [39,40,41,42], and the fast priming technique has provided similar findings [37]. This suggests that phonological recoding plays a key role in activating lexical entries during skilled adult reading; that is, a word’s phonology plays a pre-lexical role rather than a lexical/post-lexical role.

**Children.** Far less research has been done with children using research methods that are sensitive to online cognitive processing during reading. To date, though, two studies have used eye movements to examine phonological processing during children’s silent sentence reading, examining foveal reading processes. Blythe et al. [14] presented sentences containing correct target words, pseudohomophones, or orthographic controls, to both adults and children aged 7 to 9 years (e.g., *Today we had a huge water/worta/wecho fight in my back garden.*). Pseudohomophones were used due to this age group of children being limited in the number of homophone pairs known to them, especially with Age-of-Acquisition limited to earlier than 6 years to maximise the likelihood that all participants would be familiar with the target words. They found that children, similarly to adults, benefitted from the valid phonology of a pseudohomophone compared to an orthographic control. These data were argued to provide evidence for covert phonological recoding in children as young as 7 years old (contradictory to some isolated word recognition research; e.g., [22]). Two further points support this conclusion. First, all participants were reading silently, and no overt decoding was observed at any point. Clearly, these children were beyond the phase of reading development where overt decoding was their primary strategy for lexical identification. Second, and critically, when compared against reading times on the correct target word within a sentence, the cost associated with pseudohomophones was less than 200 ms, and reading times on the pseudohomophones were less than 600 ms in total in the children’s data. These reading times are too short to plausibly incorporate the sounding out and then blending together of phonemes. These data are, therefore, most consistent with phonological recoding during lexical identification, suggesting that both adults and children are able to access the correct lexical representation on the basis of a letter string’s phonology.

Moreover, Jared, Ashby, Agauas, and Levy (Experiment 3; [43]) provided further evidence that phonological representations are used in the initial activation of word meanings. The researchers monitored children’s (10 to 11 year olds) eye movements as they read sentences silently, some of which contained a correct target word (e.g., *whether*), some a homophone (e.g., *weather*), and some an orthographic control (e.g., *winter*). Critically, the homophones were not as disruptive to the children’s reading as the orthographic controls (i.e., the children displayed shorter reading times when a homophone was present than when an orthographic control was present). This observed homophone advantage reflects the contribution phonology made to activating the meanings of words for the child readers (regardless of word frequency). Phonology, therefore, seems to play a key role during children’s lexical identification during silent sentence reading. Furthermore, similar to Blythe et al. [14], the mean reading times suggest that children were undertaking phonological recoding (as opposed to overt decoding).

This research [14,43] is consistent with the view that phonology continues to play a role in aiding lexical access, but in an increasingly covert manner as age and reading skill increase [4,44]. This argument is further supported by studies that have shown increased fixation times on long words (e.g., *medicine*) compared to short words (e.g., *salt*) in both children and adults [45,46]. There are two critical points to note with respect to these studies. First, Hyönä and Olson [45] used a reading aloud task with 8–12 year old children, and no overt decoding was observed for either the long or the short words. Second, the magnitude of the increase in reading times was between 22 ms per letter ([46]; silent reading in 7–11 year old children) and 58 ms per letter [45]. The magnitude of these increases to reading times are too small to conceivably argue that children were sounding out and blending phonemes together, either vocally or subvocally, in order to achieve lexical access (phonological decoding). Both of these points support the argument that children at this age have moved beyond overt phonological decoding during lexical identification.

It is widely recognised that adults continue to make use of phonology to aid lexical access and identification during reading, e.g., [2,3], but until recently, this issue has been somewhat neglected within the empirical literature on children’s reading development. We contend that, while there is developmental change in phonological processing during reading, this is best characterised as a transition from phonological decoding to phonological recoding. Such a developmental transition is not currently accounted for in theoretical models of learning to read, which simply posit decreasing reliance on phonology as reading skill increases, e.g., [5,6,7,8,9,10].

It is worth noting that phonological processing in English, the focus of this paper, may differ from that in other languages, due to differences in orthographic depth (the consistency of a language’s GPCs). For example, English has an opaque orthography, wherein GPCs are not very consistent (i.e., *ough* in *cough*, *through*, *though*, etc.), whilst other alphabetic languages, like Finnish and German, benefit from more transparent orthographies. One piece of research has investigated phonological pre-processing in German. Tiffin-Richards and Schroeder [47] found that German adults benefitted more from orthographic than phonological information in the parafovea. Whilst children also gained some preview benefit from orthographic information in the parafovea, this was only under certain conditions: when the target words only received a single fixation and when capitalisation of the word was present. In contrast, the children did show a clear preview benefit from pseudohomophones. This would suggest that, in German, for children, phonology plays a more important role in word identification than orthography, whilst, for adults, the opposite pattern seems to occur: orthography seems to play a more dominant role in facilitating lexical access than phonology. In Chinese, a morphosyllabic language [48], phonological information has been shown to be activated pre-lexically by children, whilst adults seem to use more direct access from orthography to semantics [49]. Within Chinese, the researchers argued, early, pre-lexical activation of phonology diminishes as readers become more skilled. It is worth noting though that this research focuses on parafoveal processing of orthographic and phonological information and so does not make claims that, for instance, children do not process orthographic information foveally in German. Overall though, this research on both adults’ and children’s parafoveal pre-processing in German and Chinese seems to be in contrast to the research looking at pre-processing of phonology in English adults, e.g., [39]. Indeed, concerns have been raised as to whether research conducted in English may have overestimated the importance of phonology, e.g., [50,51]. Consequently, the developmental transition from overt, effortful phonological decoding to covert, rapid phonological recoding that appears to occur in English, as outlined in this paper, may not be applicable to other languages. Whilst phonology does seem to play a role in reading development in other alphabetic languages besides English, it does seem to be modulated by orthographic depth [51]. Evidence suggests that readers of more transparent orthographies might make the transition from phonological decoding to phonological recoding at a faster rate than readers of English, with it suggested that the difficulty associated with progressing to phonological recoding is specific to English and its complex GPCs, e.g., [52]. Thus, the extent to which reading development within different languages is determined by phonological processing may differ.

**Atypical development.** Most recently, studies have begun to show evidence for pre-lexical phonological processing in populations with atypical reading development, specifically in individuals with permanent childhood hearing loss (PCHL; [53]) and individuals with developmental dyslexia [54]. Both of these participant populations are known to commonly experience substantial difficulties in learning to read, and one component of these difficulties is thought to be poor phonological processing skills, e.g., [55,56,57]. 

In the case of individuals with PCHL, their auditory perception since birth has been substantially impoverished, and it is likely that this results in underspecified cognitive representations of phonology. Indeed, on tasks that require overt awareness of, or conscious manipulation of, speech sounds, Blythe et al. [53] found that teenagers with PCHL scored significantly lower than both chronologically age-matched and reading-matched hearing peers. Despite their difficulties in overt phonological decoding and phonological awareness, these teenagers displayed a pseudohomophone advantage both during direct fixation and from parafoveal preview. In particular, the pseudohomophone advantage shown by teenagers with PCHL was very similar in terms of both time course and magnitude to the effect observed in their younger, reading-matched hearing peers. This strongly indicates that, despite their overall difficulties in learning to read, one particular aspect of lexical processing was maturing in a typical manner (albeit with a slight developmental delay)—the transition to phonological recoding.

In the case of developmental dyslexia, both overall reading difficulties and specific difficulties in phonological awareness and processing have been well-documented; indeed, poor phonological processing skills are largely accepted as the predominant cause of developmental dyslexia, e.g., [56,57]. Again, though, recent research has shown that teenagers with dyslexia still exhibit a pseudohomophone advantage during reading during both direct fixation and parafoveal preview [54]. Similar to the data from teenagers with PCHL, this pseudohomophone advantage during silent sentence reading was observed, in contrast to significantly poorer performance on overt tasks of phonological processing compared to their typically developing peers.

In sum, eye movement research in recent years has provided strong evidence for pre-lexical phonological recoding by adults, typically developing children, and even individuals with PCHL or dyslexia during silent sentence reading, e.g., [14,37,39,40,41,42,43,53,54]. These data challenge theoretical accounts of reading development which posit that phonological processing during lexical identification reduces with time and reading skill, e.g., [5,6,7,8,9,10]. Rather, these data are more consistent with the view that, as reading skill increases, there is a transition from phonological decoding to phonological recoding. This transition seems to occur relatively early and is remarkably robust across both typical and atypical reading development.

## 5. The Role of Phonology: Models of Word Recognition

A number of different models have been put forward by researchers in attempts to explain how printed word recognition occurs (e.g., the dual-route cascaded model—DRC; [58]; the multiple-route model; [59]; connectionist dual-process model—CDP+; [60]). It is noncontroversial that all of these models posit some role for phonology in visual word recognition, but they vary in terms of the importance that is ascribed to phonology (for a recent and comprehensive review, see [43]). Here, we briefly outline these models and how each of them incorporates phonological processing into printed word identification. Critically, we consider the degree to which these models can account for developmental change in this respect.

**The DRC model.** According to this model, processing is accomplished via two distinct but interactive routes: lexical and non-lexical, see [58] (Figure 7, p. 214). The lexical (direct) route relies on the activation of word-specific orthographic representations: the features of a word’s letters activate the word’s letter units (in parallel), and these letters then activate the word’s entry in the orthographic lexicon. The non-lexical (indirect) route is based on the use of GPCs (operating serially from left to right); visual features and letter units are activated just as with the lexical route (as they are common to both routes). Processing along the direct lexical route gets faster each time a word is encountered, so the lexical representations of more common words are activated by the direct route before the slower, indirect, non-lexical route has finished processing the word, e.g., [11,23]. When tested, the DRC was 99% accurate in generating a pronunciation for the 7981 words in its orthographic lexicon. It can account for many phenomena that are observed in skilled adult reading, including frequency effects, regularity effects, the pseudohomophone advantage, and orthographic neighborhood effects. With respect to developmental change, however, the model has no learning mechanism, and “…has nothing to say about the actual process of learning to read” (p. 246). The authors argue that it does work well to characterise what a typically developing child reader has learned so far at any point during the process of learning to read, and that young readers (7 year olds) have reading systems similar to adults, albeit scaled-down versions. It is not clear, however, how the two routes would develop in a beginning reader or how the model would account for a developmental transition from decoding to recoding.

**The multiple-route model.** The multiple-route model, see [59] (Figure 2, p. 282) makes a distinction between the effortful phonological coding of beginning readers and the faster, more automatic use of phonology that develops with a reader’s exposure to print. (Note that what Grainger et al. [59] refer to as "phonological recoding" is referred to within this paper as phonological decoding). The initial, overt coding process enables the development of parallel letter processing, involving an array of letter detectors that are location-specific (i.e., that encode the locations of letters within a printed word). Two orthographic codes are generated from this: a coarse-grained route that allows direct access to semantics and a fine-grained route that codes the precise ordering of letters within a string and then activates the corresponding phonemes as well as whole-word orthography. The model clearly predicts strong, phonologically-based effects (e.g., pseudohomophone effects) in younger children that reduce but do not disappear with increasing age as the reader transitions to phonological recoding. This model, therefore, seems to be entirely consistent with the experimental observations from the body of published literature reviewed within this paper.

**The CDP+ model.** The CDP+ model [60], similar to the DRC model, has two processes: a non-lexical one (sublexical route) that links orthography to phonology and learns GPCs very quickly and a direct lexical one that links orthography to phonology, where orthographic entries are linked to their phonological counterparts (an implementation of the DRC’s lexical route). With respect to developmental change, Ziegler, Perry, and Zorzi [61] provided a computational simulation of the self-teaching hypothesis [15] within the framework of the CDP+ model. They examined the extent to which the model could learn to identify unknown words based on initial, explicit teaching of key GPC rules and its existing phonological lexicon, similar to what a child might experience. Ziegler et al. [61] argue that children receive phonics instruction early in their formal education, but they are not explicitly taught the correct pronunciation of every word that they encounter during reading. Rather, as they come across new printed word forms, they use their knowledge of phonics rules to generate a possible pronunciation and determine whether or not this matches a word that is already represented in their lexicon (through spoken language exposure). This learning loop is referred to as the phonological decoding self-teaching (PDST) hypothesis, and, indeed, the implementation of the PDST hypothesis worked in the context of a real computational model of learning to read (CDP+). Even starting with a small number of GPCs (as beginner child readers would do), the model was able to acquire word-specific orthographic representations for over 25,000 words and read novel words aloud. On the basis of these rudimentary GPCs (and decoding skills), the model could produce pronunciations for unfamiliar words. Despite the opaque orthography of English, the phonological decoding network was still able to learn up to 80% of the words. Overall, phonological decoding seems to serve as a powerful internal teaching device, as implemented in this model, allowing a basic set of GPCs to open up children’s (and the model’s) abilities to read novel words and gain orthographic knowledge. It is conceivable within the PDST hypothesis that there is a transition from beginner children’s phonological decoding to skilled adult readers’ phonological recoding, but this has not yet been operationalised.

In sum, all of these models propose that phonology plays a role in visual word recognition. To date, Grainger et al.’s [59] multiple-route model provides the clearest implementation that might account for the developmental transition from beginner child readers’ effortful phonological decoding to skilled adult readers’ unconscious, rapid phonological recoding.

## 6. Conclusions

Whilst it is widely recognised that children rely on phonological decoding in the early stages of learning to read, current theories do not fully account for skilled readers’ pre-lexical processing of phonology, that is, phonological recoding [5,6,7,8,9,10], with only one recent model of word recognition seeming to account for this developmental transition (the multiple-route model; [59]). Eye movement research has shown pre-lexical processing of phonology in typically developing readers from the age of 7 years through to skilled adult readers, as well as in atypical developmental groups, despite the tasks used not requiring any overt phonological processing [14,35,39,43,53,54]. Thus, eye movement research provides compelling evidence for phonology having a continued and pervasive role in facilitating lexical identification during reading (consistent with the multiple-route model; [59]). As such, recent empirical findings from online research methods, such as eye movement recordings, need to be incorporated into theories of learning to read, and more consideration needs to be given to these findings in developmental models of word recognition. In order to accomplish this, further research is needed to understand the nature and time course of the transition from phonological decoding to recoding, by examining moment-to-moment cognitive processing during reading in beginning readers.
